# In-Process Measurement for the Process Control of the Real-Time Manufacturing of Tapered Roller Bearings

**DOI:** 10.3390/ma11081371

**Published:** 2018-08-07

**Authors:** Francisco Javier Brosed, A. Victor Zaera, Emilio Padilla, Fernando Cebrián, Juan José Aguilar

**Affiliations:** 1 Design and Manufacturing Engineering Department, Universidad de Zaragoza, 3 María de Luna Street, Torres Quevedo Bld, 50018 Zaragoza, Spain; jaguilar@unizar.es; 2 FERSA, 18 Bari Street-PLAZA, 50197 Zaragoza, Spain; victor.zaera@fersa.com (A.V.Z.); emilio.padilla@fersa.com (E.P.); fernando.cebrian@fersa.com (F.C.)

**Keywords:** in-process measurement, geometric accuracy, grinding process, tapered roller bearings

## Abstract

Tapered roller bearings can accommodate high radial loads as well as high axial loads. The manufacturing process consists of machining processes for ring and component assembly. In this contribution, the parameters of influence on the measurement procedure were studied. These parameters of influence were classified as environmental, process, and machine parameters. The main objective of this work was to optimize the process using real-time measurements, which required the study of the influence of several parameters on the measurement uncertainty and how to correct their effects.

## 1. Introduction

Bearing manufacturing is a high-precision technology where the material composition, hardness, and micrometric dimensions need to be ensured to meet the product requirements [1,2].

The quality of the product is an important strategic factor for the competitiveness of the European manufacturing industry in the global market [3]. In this context, process control, automation, and optimization are key to having the best quality at a competitive cost [4,5]. Process control ensures quality and reduces scrap and rework, but requires dimensional measurements (the effect on the temperature of the grinding process needs to be considered). Automation is the key to achieving competitive cost by using machines with enough accuracy and cycle time, and automatic adjustment of the manufacturing process in real time. Current data collection and inspection technologies allow data to be collected online along the process chain and can significantly increase quality control and improvements in current dynamic and modifiable environments [6,7]. The real challenge facing companies is the problem of synthesizing highly heterogeneous data to gain in-depth understanding of the correlations between the variables throughout the stages of a multi-stage system. This is aimed at achieving the generation of zero defects at the single process level, and the propagation of zero defects at the system level through the proactive control of the process [8].

Tapered roller bearings can accommodate high radial loads as well as high axial loads. They have four main components: inner ring, outer ring, rollers, and cage. In general and in the case under investigation, these components are metallic, although the cage can be plastic depending on the application. The manufacturing process consists of the machining processes of rings and component assembly. The expected quality standard needs to measure each individual part using an appropriate measurement instruments. These instruments have an uncertainty in their measurements. Ambient temperature, the temperature of the system, and the temperature of the part under inspection also influences the precision of the components and of the mounted bearing [9]. Different methods can be used to measure the size of the parts: touch probes [10,11], air pressure [12,13], and laser systems [14,15]. Most of the developments that can be found in the literature regarding bearing inspection are methods for fault diagnosis and service life estimation. Many experiments and studies have been performed to explore the nature of bearing defects with the help of several monitoring techniques such as vibration, acoustic emissions, oil-debris, ultrasound, electrostatic, shock-pulse measurements, etc. [1,16,17], although some of them estimate the size of the manufacturing defects of tapered roller bearings with vibration measurement [18].

As stated before, process optimization and online monitoring and control are key factors for improving efficiency and quality in machining [19]. With the development of intelligent machining, the optimization of cutting process configuration during actual production has become more accessible, and optimizing the volume of removed metal by adjusting the grinding time for each part can decrease the process time and improve tool life [20,21]. Finally, the grinding machine control system can receive the measurement results of the machined part as feedback information for process verification.

**Figure 1:**
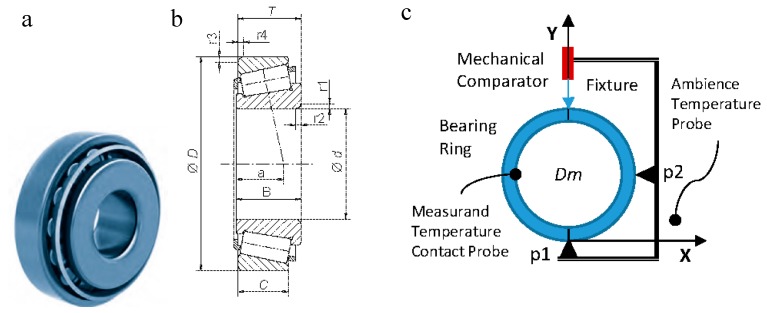

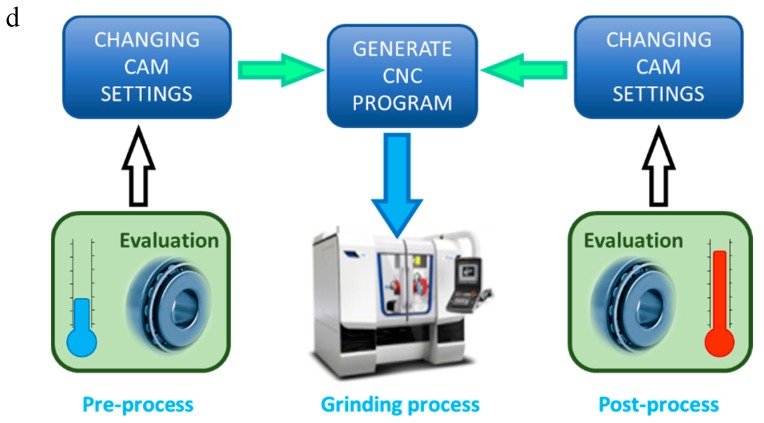
(**a**) Tapered roller bearing; (**b**) Functional dimensions; (**c**) Measurement device; (**d**) Synoptic of operation.

The work described in this article focused on the “in process” verification [22,23] of a tapered roller bearing. The magnitude under inspection was the outside diameter of the outer ring (*D* in Figure 1). Its design tolerance was ±0.025 mm. The measurement result was needed to feedback the real-time control of the grinding process used to manufacture the outside diameter of the outer ring. The authors describe the model and analysis of a measurement system and the effects of its main error sources, namely the temperature and the misalignment of the devices or of the work piece and the master piece. The influence of the error sources was studied and an estimation of the uncertainty of the system was provided using simulations programmed using the Monte Carlo method [24,25], and finally the process improvement achieved when the measurement results were fed back into the manufacturing process is shown. A general approach for modelling the uncertainty associated with coordinate measuring systems (CMSs) is given in [26]. Several authors [24,25] have shown a comparison between the estimation of measurement uncertainty using the law of propagation of uncertainty [27] and that using the propagation of distribution using the Monte Carlo method [28]. In this case, the method presented in [28] was the one that better fit our application, as the variables affecting the results of the measurement presented different distributions, some of them with asymmetrical effects. A comparison of the results obtained with the simulation using the Monte Carlo method and the experimental results allowed for the identification of the main error sources and quantified their influence.

## 2. Materials and Methods

The measurement of the diameter of the outer ring (“*D*” in Figure 1) was carried out using a mechanical comparator, a registration tool to position the ring, and two temperature probes, one per contact to measure the temperature of the piece and another to measure the ambient temperature. A scheme of the system taking the measurement appears in Figure 1 and the characteristics of its components are indicated in Table 1.

The tooling was adjusted using a standard part (or master). The adjustment of the tooling consisted of fixing the position in the X direction of the support point p2 (Figure 1). For this purpose, a master piece was used, the previously-calibrated diameter of which corresponded to the nominal diameter to be measured with the system (in this case *D*_0_ = 112.712 mm). The point p2 shifted in the X direction until the maximum indicated by the probe was obtained. In this position, p2 was set. This operation was carried out each time the reference was changed.

Once the tooling had been adjusted, the zero of the comparator with the diameter of the masterpiece (*D*_0_) was set as the reference. This operation was performed every 30 min (approximately every 50 parts checked). Both the machine and the masterpiece were stabilized at room temperature as the master piece was kept in the workshop between measurements.

After these two steps, the system was ready to measure the diameter of the outer rings (*D_m_*) after being rectified so that its temperature (*T_m,m_*) was higher than the ambient temperature at the time of performing the measurement (*T_a,m_*). The verification of the outer ring was performed at a rate of approximately 100 parts/h. The system and the masterpiece were kept in the workshop, thus it was considered that both elements were at room temperature (Equations (1) and (2)).

*T*_*m*,0_ = *T*_*s*,0_ = *T*_*a*,0_ (1)

*T_s,m_* = *T_a,m_* (2)

where *T_x,t_* is the temperature of the ambience if *x* = *a*, of the system if *x* = *s*, and of the measured part if *x* = *m*. The temperature changes over time, thus *T_x,t_* indicates the temperature at the moment of measuring the *m*-th part if *t* = *m* and at the time of measuring the master piece if *t* = 0.

The following system elements were introduced in the model of the measuring system: *D_m_*, measurand; *H*, tooling height; and *L_m_*, probe length in the measurement of the *m*-th piece. The influence factors considered and the indicated system elements are summarized in Table 2 together with the nomenclature used in this work.

Factors due to the measurement process were considered, such as the possible inclination of the part due to incorrect support in the tooling (*θ_m_*). This angle depends on the measurement process and therefore may be different when measuring the master piece and the part to be checked.

Finally, we analyzed the effect of factors influencing the configuration and mechanical behavior of the system. Therefore, the effect on the result of the measurement of a possible displacement of the mechanical comparator (*λ_m_*) and the effect of an angular deviation of the probe from the vertical (*ψ_m_*) was studied. It should be noted that when it comes to system factors, it is reasonable to consider that its values will not be modified between the measurement of the master piece and the measurement of the rest of the parts, thus their effect will tend to cancel out, as will be verified later (Section 3.3).

The following sections analyze the effect of these factors in detail. The measurement model used in the mentioned analysis is explained hereafter:

The master piece is measured at a different temperature than the *m*-th part. In this way, it can be said that in measuring the master at *T_m,_*_0_, a diameter *D*_0,0_ (Equation (3)) is measured and the mechanical comparator at temperature *T_s,_*_0_ provides a reading *L*_0_ (corresponding to a length of the probe *L*_0,0_) (Equation (4)).

(3) $$\;D_{0,0}=D_{0}\cdot \left(1+\alpha_{0}\left(T_{a,0}-20\right)\right)\;$$

(4) $$\;L_{0,0}=L_{0}\cdot \left(1+\alpha_{L}\left(T_{a,0}-20\right)\right)\;$$

In addition, since there is a slow variation in plant temperature, both the tooling-comparator system and the master piece are considered at plant temperature, *T_s,_*_0_ = *T_m_*_,0_ = *T_a_*_,0_ (Equation (1)). At this point, the length of the comparator for which the system reference is defined can be written according to the height of the tooling, *H_s_*_,0_ (Equation (5)), depending on the nominal value and its temperature *T_s,_*_0_ (Equation (6)).

(5) $$\;L_{0,0}=H_{s,0}-D_{0,0}\;$$

(6) $$\;H_{s,0}=H\cdot \left(1+\alpha_{s}\left(T_{a,0}-20\right)\right)\;$$

The measurement of the *m*-th part is performed through a comparison with the master piece. The temperature of the part will be *T_m,m_* and that of the equipment *T_s,m_*, which is taken as the ambient temperature at the time of inspection (Equation (2)). When measuring the diameter *D_m,m_* (Equation (7)), the comparator will provide a reading Δ*L_m_* (Equation (8)) (corresponding to a length of the probe *L_m,m_* (Equation (9)).

(7) $$\;H_{s,m}=H\cdot \left(1+\alpha_{s}\left(T_{a,m}-20\right)\right)\;$$

(8) $$\Delta L_{m,m}=\Delta L_{m}\cdot \left(1+\alpha_{s}\left(T_{a,m}-20\right)\right);$$

(9) $$\;\Delta L_{m,m}=L_{0,m}-L_{m,m};\;$$

where *α_x_* is the thermal expansion coefficient of the *m*-th part if *x* = *m*; of the master piece if *x* = 0; and of the measuring system and the probe if *x* = *s*. In this case, *α_m_* = *α*_0_ = *α_s_* = 11 × 10^−6^ °C^−1^.

Substituting Equation (9) into Equation (7) provides the dimension of the diameter (Equations (10) and (11)).

(10) $$\;D_{m,m}=H_{s,m}-L_{0,m}-\Delta L_{m,m}\;$$

(11) $$\;D_{m,m}=D_{m}\cdot \left(1+\alpha_{m}\left(T_{m,m}-20\right)\right);\;$$

*L*_0*,m*_ can be calculated by taking the reference each time the temperature changes (Equation (12)). Substituting Equation (12) into Equation (10), the part diameter from the master piece data and the probe reading is calculated (Equation (13)).

(12) $$L_{0,m}=H_{s,m}-D_{0,m};\;\mathrm{with}\;D_{0,m}=D_{0}\cdot \left(1+\alpha_{0}\left(T_{a,m}-20\right)\right)$$

(13) $$D_{m,m}=H_{s,m}-H_{s,m}-D_{0,m}-\Delta L_{m,m}\Rightarrow \;\Rightarrow D_{m,m}=D_{0}\cdot \left(1+\alpha_{0}\left(T_{a,m}-20\right)\right)-\Delta L_{m}\cdot \left(1+\alpha_{s}\left(T_{a,m}-20\right)\right)$$

**Figure 2:**
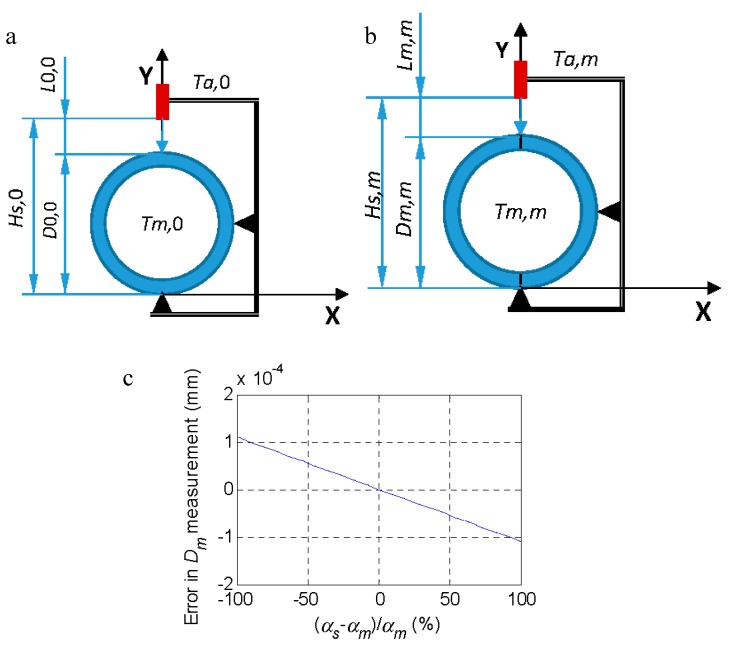
(**a**) Definition of the reference with *D*_0_ at temperature *T_m,_*_0_ = *T_a,_*_0_. (**b**) comparison with the part under inspection *D_m_* at temperature *T_m,m_* where the ambient temperature is *T_a,m_*. (**c**) Error (15)–(13) as a function of the difference between *α_s_* and *α_m_* for a temperature *T_m,m_* = 30 °C and *T*_0,0_ = 20 °C.

In the case of not measuring the reference when the temperature changes, it is possible to estimate *L*_0,*m*_ (Equation (14)) and *D*_0,*m*_ (Equation (15)), however, the result of Equations (13) and (15) will only coincide if the thermal expansion coefficients of the probe and the measurand coincide. In Figure 2, the error of Equation (15) calculated as Equations (15)–(13) is plotted when *α_s_* differs from *α_m_*.

(14) $$\;L_{0,m}=L_{0}\cdot \left(1+\alpha_{L}\left(T_{a,m}-20\right)\right)\;$$

(15) $$\;D_{m,m}=H_{s,m}-L_{0}\cdot \left(1+\alpha_{0}\left(T_{a,m}-20\right)\right)+\Delta L_{m}\cdot \left(1+\alpha_{s}\left(T_{a,m}-20\right)\right)\;$$

To obtain *D_m_*, the mechanical comparator reading for the *m*-th part was compared with the reading taken when measuring *D*_0_, thus the result of the measurement (*D_m_* at 20 °C) was obtained from Equation (16).

(16) $$D_{m}=\frac{H\cdot \left(1+\alpha_{s}\left(T_{a,m}-20\right)\right)-L_{0}\cdot \left(1+\alpha_{L}\left(T_{a,m}-20\right)\right)+\Delta L_{m}\cdot \left(1+\alpha_{L}\left(T_{a,m}-20\right)\right)}{1+\alpha_{m}\left(T_{m,m}-20\right)}\;$$

## 3. Results

### 3.1. Effect of Temperature on Measurement

The effect of the temperature in the process can be corrected using Equation (16), as the temperature was known. However, if one of the process temperatures (the ambient temperature or the temperature of the *m*-th part) was not known, the following situations could occur, as described in Table 3.

**Figure 3:**
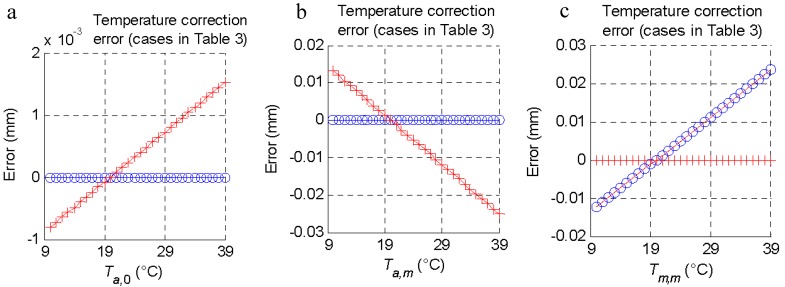

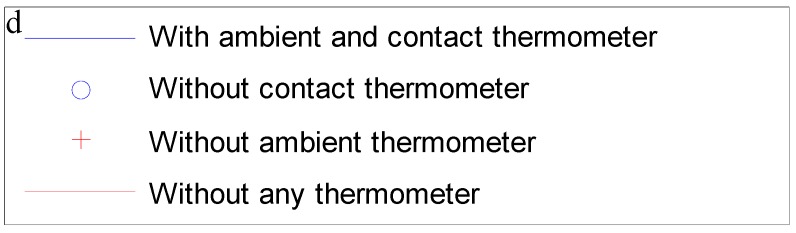
Temperature compensation depending on the measurement process: correction error of the cases described in Table 3 (the first case of Table 3 is the reference); (**a**) Effect of the ambient temperature variation at the moment of measuring the masterpiece (*T_a,_*_0_). (**b**) Effect of the ambient temperature variation at the moment of inspecting the *m*-th part (*T_a,m_*). (**c**) Effect of the temperature of the *m*-th part (*T_m,m_*) variation. (**d**) Legend of the results from using each case of Table 3.

The effect of the ambient temperature and the temperature of the *m*-th part was studied by varying them independently and evaluating the error (Equation (11)) that they introduced to the calculation of the diameter in the function of the four cases raised (Table 2). In each case, a variation between 10 and 39 °C was introduced for the ambient temperature at the moment of measuring the masterpiece (*T_a,_*_0_), the ambient temperature at the moment of inspecting the *m*-th part (*T_a,m_*), and the temperature of the *m*-th part (*T_m,m_*) (Figure 3). From these results, it was shown that by applying Equation (16), it was possible to measure at any temperature. However, if one of the two probes was not available, an error was introduced in the measurement. For these cases, the temperature increase necessary to obtain a measurement error equal to the tolerance (±0.025 mm) was quantified, and is presented in Table 2. As can be seen, the influence of the ambient temperature at the moment of taking the reference with the master piece had less weight than temperatures at the time of measuring the *m*-th part.

### 3.2. Effect of Process and Machine Factors on the Measurement

**Figure 4:**
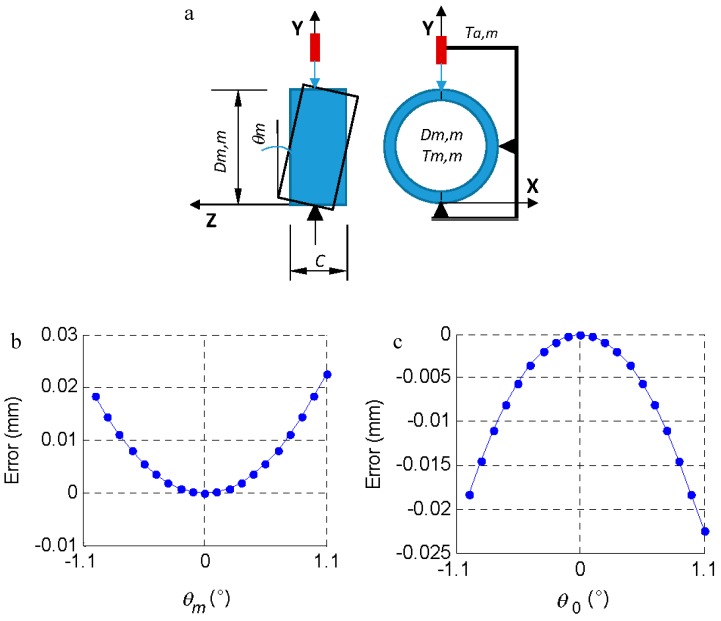
(**a**) *θ_m_*, inclination of the piece when it is placed in the measuring tool. (**b**) Effect of the inclination of the *m*-th part (*θ_m_*) on the measurement error (comparator reading—*D_m,m_*). (**c**) Effect of the inclination of the masterpiece (*θ*_0_) on the measurement error (comparator reading—*D*_0,0_).

**Figure 5:**
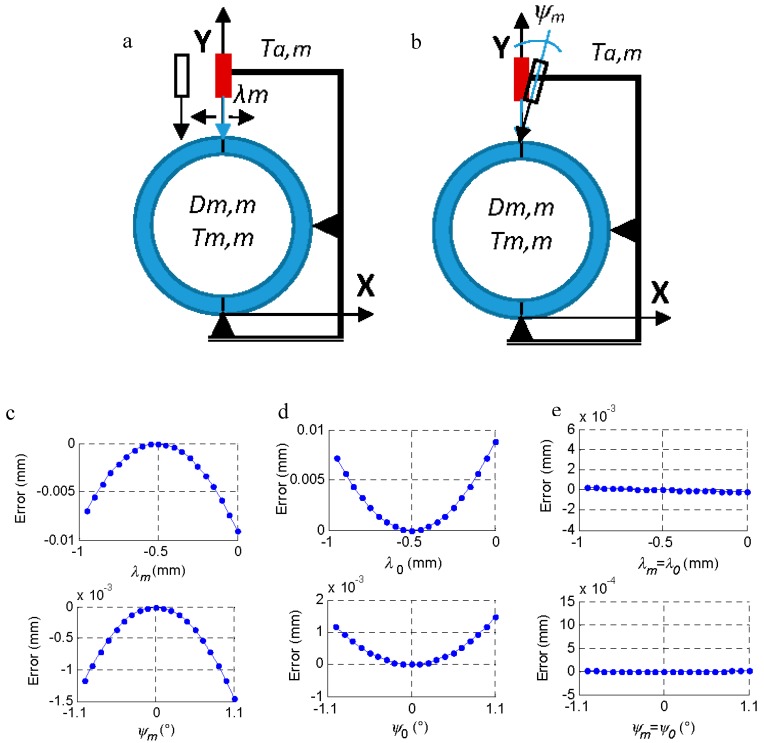
Effect of the system deformation on the measurement error. (**a**) Moving the probe *λ_m_*. (**b**) Turning the probe *ψ_m_*. (**c**) Effect of *λ_m_* and *ψ_m_* on the measurement of *D_m,m_*. (**d**) Effect of *λ*_0_ and *ψ*_0_ on the measurement of *D_m,m_*. (**e**) Effect if *λ_m_* = *λ*_0_ and *ψ_m_* = *ψ*_0_ in the measurement of *D_m,m_*.

In this section, we analyzed the effect of the incorrect positioning of the part on the tooling (*θ_m_*, process factor) and the effect of a possible deformation of the tooling-comparator system (*λ_m_* displacement and rotation *ψ_m_*, machine factors) (Figure 4 and Figure 5).

The effect of improper support on the tooling was evaluated, thus a sloping part (*θ_m_*) was measured instead of measuring a part that was perfectly horizontal (Figure 4). This can occur both when inspecting the *m*-th part (*θ_m_*) and when measuring the master piece (*θ*_0_) and may be due, for example, to the presence of a chip in the machining process. The geometry of the piece (dimension *C* = 23.812 mm) limits the maximum inclination (11°) from which the measurement would not be possible. In the measurement process, a slope close to the indicated maximum would be visually detected, so the slope range studied was lower (± 1.1°, Figure 4b,c).

If the part is measured with a slope of ± 1.1°, the measurement error (comparator reading—*D_m,m_*) is 0.018 mm.

If the machine suffers some type of deformation, the probe may suffer shifts and rotations that cause incorrect measurement (Figure 5a,b). A displacement (*λ_m_*) on the X axis prevents correct probing at the diametrical point of the part, and a rotation (*ψ_m_*) of the probe on the Z axis introduces a cosine error in the probe reading. These effects were introduced independently in the measurement of the master piece (*λ*_0_ and *ψ*_0_) and the measurement of the *m*-th part (*λ_m_* and *ψ_m_*), but, as is reasonable to assume, if the system is deformed in the measurement of the master piece, it will also be so in the part, and the effect is the same as in Equations (17) and (18), Figure 5.

*λ_m_* = *λ*_0_, (17)

*ψ_m_* = *ψ*_0_, (18)

The effect of *θ_m_* is inverse to that of *λ_m_* and *ψ_m_* since *θ_m_* tends to increase the reading when its value increases, while *λ_m_* and *ψ_m_* tend to increase the length of the probe when taking the reading and, therefore, reduce the diameter of the ring when they increase their value, as seen in Figure 5. The effect of *λ_m_* and *ψ_m_* tends to be canceled when their values are the same at the time of measurement of the master piece and at the time of measurement of the rest of the parts (Equations (17) and (18), Figure 5e).

### 3.3. Estimation of the Uncertainty and Contribution of Each Parameter

Simulations were programmed using the Monte Carlo method [24,25] to determine the contribution of each parameter to the final uncertainty. The probability density distributions (PDD) of each factor were defined based on its calibration data or the characteristics of the process. The PDDs associated with the measurement equipment, such as temperature probes or the mechanical comparator, were defined based on their measurement uncertainty. The PDDs associated with the process or tooling factors were defined from a uniform distribution where the limit values were defined from the analysis performed in Section 3 and according to the process and characteristics of the tooling (Table 4).

**Figure 6:**
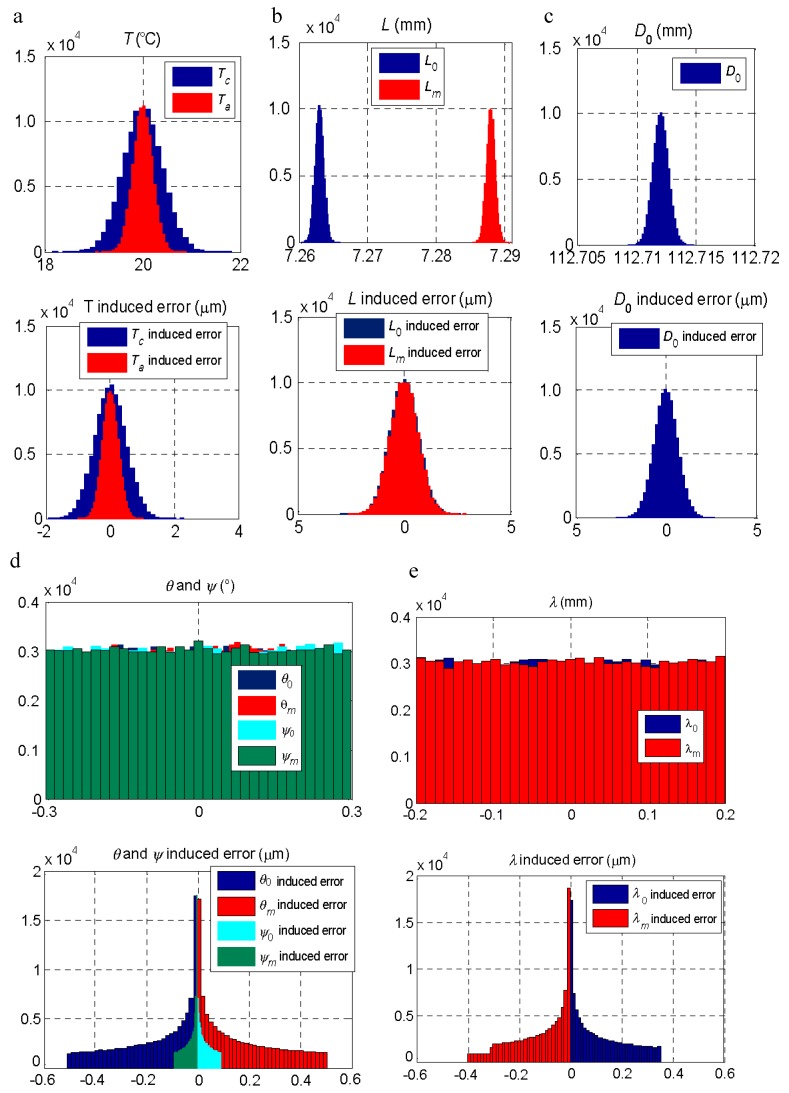
Influence of the variability of the factors on the measurement result. Above, variation introduced in the parameter. Below, the error resulting from the measurement when entering the variation of each parameter. (**a**) Ambience (*T_a_*) and measurand (*T_m_*) temperature. (**b**) Probe length when measuring the master piece (*L*_0_) and the measurand (*L_m_*). (**c**) Master piece diameter (*D*_0_). (**d**) System angles, *θ* and *ψ*, when measuring the master piece (*θ*_0_ and *ψ*_0_) and the measurand (*θ_m_* and *ψ_m_*). (**e**) System length, *λ*, when measuring the master piece (*λ*_0_) and the measurand (*λ_m_*).

A simulation of the effect of each parameter was carried out by introducing the PDDs indicated in Table 4. The results of the simulation of the effect of each parameter are shown in Figure 6, where the distribution of the error in the measurement resulting from the variation of the parameter below it are presented.

**Figure 7:**
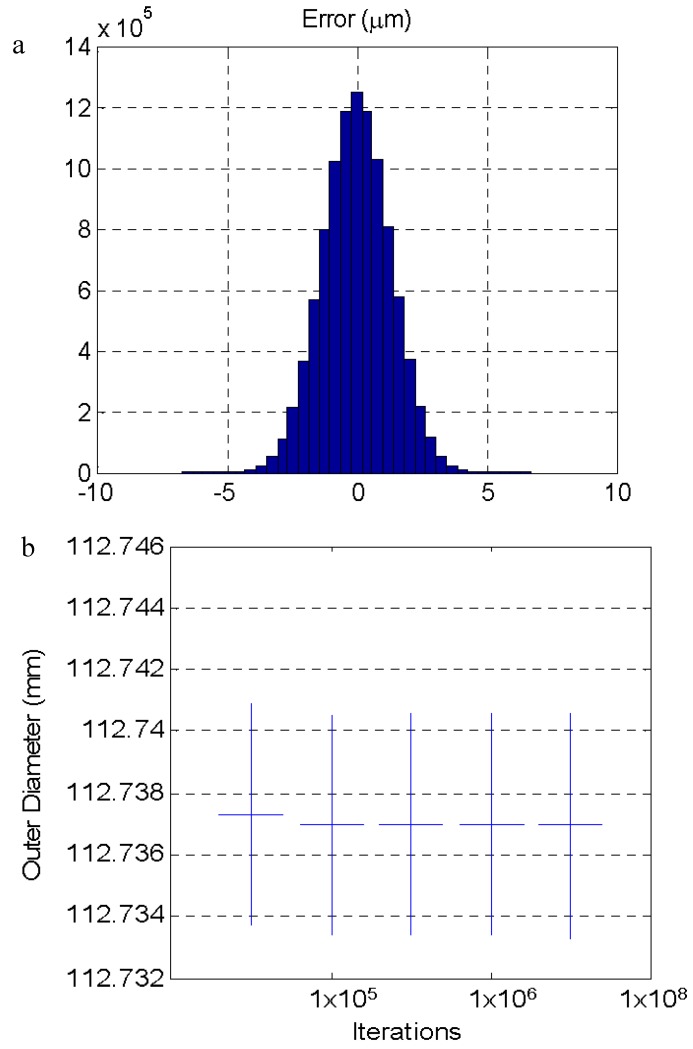
(**a**) Distribution of the error by combining all analyzed parameters with their corresponding distributions. (**b**) Convergence of the result of the simulation.

**Figure 8:**
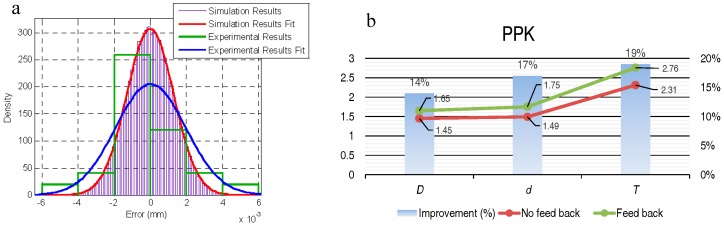
(**a**) Comparison between the results obtained using the Monte Carlo simulation and those obtained experimentally. (**b**) PPK results after applying the process measurement methodology modeled in this work for the external diameter of the outer ring of the bearing (*D*); and results obtained after applying this methodology to the internal diameter of the inner ring (*d*) and to the thickness (*T*). “*D*”, “*d*” and “*T*” according to Figure 1b.

Simulation using the Monte Carlo method allows for the estimation of the value of the measurement uncertainty according to the “Guide to the expression of uncertainty in measurement” (GUM) and its supplement 1 [28] from the PDD of the influence factors. From the simulation shown in Figure 7, the uncertainty values shown in Table 4 were obtained as a function of the parameter that introduced the variation. At the end of Table 4, the uncertainty obtained when combining the variation of all the factors appeared. The distribution of the error by combining all the factors is shown in Figure 8 together with the evolution of the results of the uncertainty estimated as a function of the number of iterations used. The result stabilized from 10^5^ iterations. Other authors [24,29,30] have observed a similar number of iterations to obtain a stabilized result with simulation using the Monte Carlo method.

## 4. Discussion

The uncertainty obtained through simulation using the Monte Carlo method can be compared with the variability of the experimental results. The contribution of each error source can be extracted from the results presented. In this way, the uncertainty in the measurement of the measurand temperature and that in the measurement of the probe length with the comparator dial are the error sources with the main effect in the final uncertainty. The results obtained from the simulation also allowed us to estimate the number of iterations needed to obtain a stable result of the uncertainty. In this case, if the number of iterations is greater than 10^5^, then the value of the uncertainty is stable.

In general, the greater the variability of a factor, the greater its contribution will be to the final uncertainty. For this reason, the variability assigned to each variable affecting the result was taken from the calibration certificate of the instrument, this was the case for the thermometers and the comparator dial, or from an analysis of the geometrical characteristics of the part under inspection and the system. This was the case for the variables of the process and the measurement system, where a study of the possible inadequate use of the system or defect occurrence in the measurand was made.

The dispersion of the experimental results (standard deviation: 0.0019 mm) was similar to that obtained with the simulation by the Monte Carlo method (standard deviation: 0.0018 mm) although the distribution of the values in the experimental results was not as close to a normal distribution as it was to the distribution obtained with the simulation (Figure 8a). From these results, the effect of the temperature on the measurement process and the need to control or monitor it to avoid an increase of the measurement uncertainty was clear, and its influence and the influence of the other variables was quantified.

When applied to the machining process of tapered roller bearings, the process measurement methodology modeled in this work for the external diameter of the outer ring of the bearing and the feedback of the measurement results showed an improvement in process capability (PPK) and a 90% reduction of rework/scrap. This methodology was tested with other dimensions of the bearing. Figure 8b represents the results obtained after applying this methodology to the internal diameter of the inner ring, *d*, and to the thickness, *T* (in addition to *D*, the diameter, the measurement of which was the object of this work).

## 5. Conclusions

From the results, it can be concluded that the values obtained experimentally and those obtained by simulation presented a similar dispersion. The Monte Carlo simulation, instead of being a large process, provided reliable results and was a useful method to identify the error sources and quantify their influence by taking into account the correlation between each source (this point could be especially complicated by applying the law of propagation of uncertainty that is also explained in the GUM [27]). Thus, when calculating the uncertainty of a system that has a complex mathematical model, it is preferable to apply Monte Carlo simulation if we can detect and quantify the significant sources that affect the system.

More generally, it can be added that the implemented methodology allows for complex and real-time control of the precision manufacturing process by means of contact sensors and temperature probes, in this case, for dimensions of the tapered roller bearing like “*D*” and “*d*”. These measurements are able to control the grinding process in real time and improve the final part quality, scrap and rework, and reduce costs by optimizing the cycle time.

## Figures and Tables

**Table 1:** Components of the measurement system.

Equipment	Range	Resolution	Expanded Uncertainty (*k* = 2)
Mechanical comparator (probe)	−1.5/+1.5 mm	0.001 mm	0.0013 mm
Contact probe thermometer	0 a 250 °C	0.1 °C	0.39 °C
Thermocouple probe thermometer	−50 a 100 °C	0.1 °C	0.40 °C

**Table 2:** Factors influencing the measurement result and system elements. Nomenclature.

Influencing Factors				Measurement System Elements (H, L, D)											
*T*, temperature (°C)	*T_x,t_*	*t* = *m*	*t* = 0	*H*, tooling height (mm)	*H_s,t_*	*t* = *m*	*t* = 0	*L*, probe length (mm)	*L_x,t_*	*t* = *m*	*t* = 0	*D*, measurand (mm)	*D_x,t_*	*t* = *m*	*t* = 0
	*x* = *a*	*T_a,m_*	*T_a,_*_0_			*H_s,m_*	*H_s,_*_0_		*x* = *m*	*L_m,m_*	N.a. ^(1)^		*x* = *m*	*D_m,m_*	N.a. ^(1)^
	*x* = *s*	*T_s,m_*	*T_s,_*_0_						*x* = 0	*L*_0,*m*_	*L*_0,0_		*x* = 0	*D*_0,*m*_	*D*_0,0_
	*x* = *m*	*T_m,m_*	*T_m,_*_0_		*H*, tooling height at 20 °C				*L_t_*, probe length at 20 °C				*D_t_*, measurand at 20 °C		
*θ_t_* (°)		*θ_m_*	*θ*_0_												
*ψ_t_* (°)		*ψ_m_*	*ψ*_0_		-	*H*			-	*L_m_*	*L*_0_		-	*D_m_*	*D*_0_
*λ_t_* (mm)		*λ_m_*	*λ*_0_												

*x*, parameter corresponding to the measurand, *x* = *m*; to the system, *x* = *s*; to the ambience, *x* = *a*; and to the master piece, particular case of measurand, *x* = 0. *t*, value of the parameter during the measurement of the *m*-th part, *t* = *m*; and of the master piece, particularly in the case of measuring, *t* = 0. ^(1)^ N.a.: Not applicable.

**Table 3:** Cases if one or both of the temperature probes are not available. “1” means that the temperature data are available, “0” means that the temperature data are not available.

Diameter (eq.)	*T_a_*	*T_c_*	Δ*T* for 0.025 mm Error (°C)
*D_m_* with ambient and contact thermometer (16)	1	1	Not applicable
*D_m_ w*/*o* contact thermometer	1	0	20.8
*D_m_ w*/*o* ambient thermometer	0	1	19.6
*D_m_ w*/*o* any thermometer	0	0	19.6

**Table 4:** Distribution and range of variation assigned to each parameter. Estimated uncertainty in each case (for each parameter and at the end of the table for all parameters at a time).

Simulation Input Parameters			Simulation (10^6^ Iterations) by Varying a Single Input Parameter		
Parameter	Equipment	Distribution	Measurement Uncertainty Um (*k* = 2)	Up. Limit	Low. Limit
*T_c_* (°C)	Contact Therm.	Normal (*µ* = 30; *σ* = 0.39/2)	0.0014	112.7356	112.7384
*T_a_* (°C)	Ambient Therm.	Normal (*µ* = 20; *σ* = 0.40/2)	0.0007	112.7363	112.7377
*Lm*, *L*_0_, *D*_0_ (mm)	Mech.probe	Normal (*µ* = 0; *σ* = 0.0013/2)	0.0018	112.7352	112.7389
*θ_m_* (°)	Parameters of the measurement process and of the system whose variation is estimated with a uniform distribution	Uniform (U.L. = −0.003; L.L. = 0.003)	0.0003	112.7365	112.7370
*θ*_0_ (°)			0.0003	112.7370	112.7375
*ψ_m_* (°)		Uniform (U.L. = −0.005; L.L. = 0.005)	0.0000	112.7370	112.7371
*ψ*_0_ (°)			0.0000	112.7369	112.7370
*λ_m_* (mm)		Uniform (U.L. = −0.2; L.L. = 0.2)	0.0002	112.7370	112.7373
*λ*_0_ (mm)			0.0002	112.7366	112.7370
Simulation (10^6^ iterations) varying all parameters			0.0036	112.7334	112.7406

## Nomenclature

*T_a,m_* (°C)	Ambient temperature when measuring the *m*-th piece.
*T_a,_*_0_ (°C)	Ambient temperature when measuring the master piece.
*T_s,m_* (°C)	System temperature when measuring the *m*-th piece.
*T_s,_*_0_ (°C)	System temperature when measuring the master piece.
*T_m,m_* (°C)	Measurand temperature when measuring the *m*-th piece.
*T_m,_*_0_ (°C)	Measurand temperature when measuring the master piece.
*T_c_* (°C)	Temperature from the contact thermometer (used in the Monte Carlo simulation).
*T_a_* (°C)	Temperature from the ambient thermometer (used in the Monte Carlo simulation).
*H_s,m_* (mm)	Tooling height of the system (see Figure 2) when measuring the *m*-th piece.
*H_s,_*_0_ (mm)	Tooling height of the system (see Figure 2) when measuring the master piece.
*H* (mm)	Tooling height of the system at 20 °C.
*L_m,m_* (mm)	Probe length when measuring the *m*-th piece.
*L*_0*,m*_ (mm)	Probe length from the measurement of the master piece but when measuring the *m*-th piece.
*L*_0*,*0_ (mm)	Probe length when measuring the master piece.
*L_m_* (mm)	Probe length when measuring the *m*-th piece (dimension at 20 °C).
*L*_0_ (mm)	Probe length when measuring the master piece (dimension at 20 °C).
*D_m,m_* (mm)	Measurand (diameter) of the *m*-th piece at the moment of measure it.
*D*_0*,m*_ (mm)	Measurand (diameter) of the master piece at the moment of measure the *m*-th piece.
*D*_0*,*0_ (mm)	Measurand (diameter) of the master piece at the moment of measure it.
*D_m_* (mm)	Measurand (diameter) of the *m*-th piece (dimension at 20 °C).
*D*_0_ (mm)	Measurand (diameter) of the master piece (dimension at 20 °C).
*ΔL_m_* (mm)	Figure, at 20 °C, of the difference between *L*_0,*m*_ and *L_m,m_*, see Equations (8) and (9).
*θ_m_* (°)	Angle between a vertical line and the front face of the ring (see Figure 4) when measuring the *m*-th piece.
*θ*_0_ (°)	Angle between a vertical line and the front face of the ring (see Figure 4) when measuring the master piece.
*ψ_m_* (°)	Angle between a vertical line and the axis of the contact prober of the comparator dial when measuring the *m*-th piece (see Figure 5).
*ψ*_0_ (°)	Angle between a vertical line and the axis of the contact prober of the comparator dial when measuring the master piece (see Figure 5).
*λ_m_* (°)	Linear translation on X-axis direction (see Figure 5) when measuring the *m*-th piece.
*λ*_0_ (°)	Linear translation on X-axis direction (see Figure 5) when measuring the master piece.
*α_m_* (°C^−1^)	Thermal expansion coefficient of the *m*-th piece.
*α*_0_ (°C^−1^)	Thermal expansion coefficient of the master piece.
*α_s_* (°C^−1^)	Thermal expansion coefficient of the system.
